# Supplementary material for: Baseline Characterization of the Gut Microbiota of Field and Colony Populations of Phlebotomus tobbi and Preliminary Assessment of the Anti-Leishmanial Activity of Cultivable Bacteria
Source: Pathogens. 2026 Jun 23;15(7):658. doi: 10.3390/pathogens15070658 (PMC13416292; doi:10.3390/pathogens15070658)
Supplement: Supplementary file 1 [file pathogens-15-00658-s001.zip › pathogens-4357168-File S1/Taxa2ASV_Decomposer/Family/f__Sphingomonadaceae/seqs.mltree.pdf]

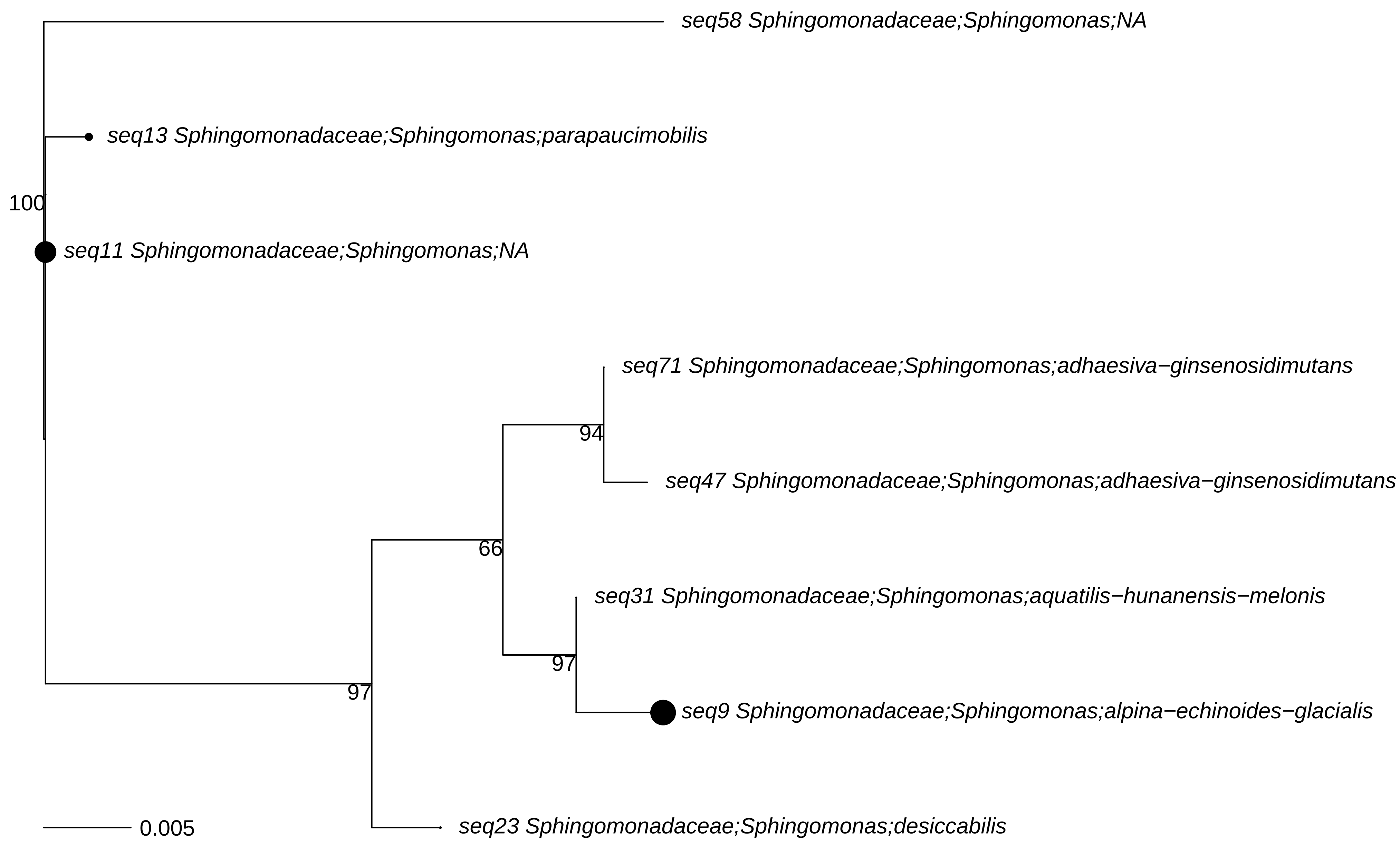
seq58 Sphingomonadaceae;Sphingomonas;NA
seq13 Sphingomonadaceae;Sphingomonas;parapaucimobilis
100
seq11 Sphingomonadaceae;Sphingomonas;NA
seq71 Sphingomonadaceae;Sphingomonas;adhaesiva−ginsenosidimutans
94
seq47 Sphingomonadaceae;Sphingomonas;adhaesiva−ginsenosidimutans
66
seq31 Sphingomonadaceae;Sphingomonas;aquatilis−hunanensis−melonis
97
97
seq9 Sphingomonadaceae;Sphingomonas;alpina−echinoides−glacialis
0.005
seq23 Sphingomonadaceae;Sphingomonas;desiccabilis
